# Cross-Partner Associations of Female Reproductive Hormones With Semen Parameters in Unexplained Infertility: An Exploratory Secondary Analysis

**DOI:** 10.7759/cureus.117329

**Published:** 2026-10-01

**Authors:** Ikran Mohamud, Mena Abdalla

**Affiliations:** 1 Obstetrics and Gynaecology, Princess Royal University Hospital, London, GBR; 2 Medical Education, Queen's University Belfast, Belfast, GBR; 3 Obstetrics and Gynaecology, King's College Hospital NHS Foundation Trust, London, GBR

**Keywords:** female, female infertility, hormonal, infertility, male, semen

## Abstract

Background

Unexplained infertility is a couple-level diagnosis made after standard female and male investigations do not identify a specific cause. Subtle cross-partner reproductive patterns may nevertheless be present. This exploratory secondary analysis assessed associations between female reproductive hormones and partner semen parameters in couples with unexplained infertility.

Methods

This cross-sectional secondary analysis used paired data from 70 couples with unexplained infertility from a previously published case-control study conducted at Ain Shams University Maternity Hospital in Cairo, Egypt, from October 2018 through April 2019. The original study evaluated vitamin D and reported descriptive hormone and semen data; the cross-partner analyses reported here are new. Female follicle-stimulating hormone, luteinizing hormone, prolactin, estradiol (E2), and thyroid-stimulating hormone (TSH) were correlated with partner semen volume, concentration, total count, total motility, progressive motility, and normal morphology using Spearman correlation. Thirty correlations were screened with Benjamini-Hochberg false-discovery-rate (FDR) adjustment. Targeted linear models were adjusted for female age and body mass index (BMI), with 5,000-resample bootstrap confidence intervals.

Results

Female prolactin was inversely correlated with partner progressive motility (rho = -0.472, FDR q < 0.001) and normal morphology (rho = -0.595, FDR q < 0.001). TSH correlated positively with progressive motility (rho = 0.331, FDR q = 0.038) and morphology (rho = 0.419, FDR q = 0.003). E2 was nominally correlated with sperm concentration (rho = 0.284, p = 0.017) but did not survive FDR adjustment (q = 0.103). In age- and BMI-adjusted models, prolactin remained inversely associated with progressive motility (B = -0.401, bootstrap 95% CI -0.687 to -0.147) and morphology (B = -0.929, bootstrap 95% CI -1.282 to -0.620). The adjusted TSH association with morphology persisted (B = 4.076, bootstrap 95% CI 0.250 to 8.016), whereas the bootstrap interval for progressive motility included the null.

Conclusions

Female prolactin showed the most consistent inverse couple-level association with partner progressive motility and normal morphology. These cross-sectional findings do not establish biological effects of female hormones on male semen quality and should be regarded as hypothesis-generating pending independent prospective replication.

## Introduction

Infertility is assessed at the level of the couple, with concurrent evaluation of female and male factors. Unexplained infertility is a diagnosis of exclusion after standard female and male investigations do not identify a specific, treatable cause [1,2]. Contemporary male-infertility guidance emphasizes evaluation of the male partner, including reproductive history, examination, and semen analysis [3]. Standardized semen examination remains fundamental to that assessment [4]. However, the diagnostic limits of conventional semen parameters and the evidence gaps in male-infertility assessment require that results be interpreted within the clinical context of the couple rather than as standalone measures of fertility potential [5-7].

Prospective couple studies indicate that some semen parameters are associated with time to conception at the population level, yet they do not create a single definitive threshold that separates fertile from infertile couples [8,9]. Accordingly, unexplained infertility does not exclude subtle reproductive characteristics or coupled exposures that may jointly influence fertility potential.

Female reproductive hormones reflect aspects of ovarian and pituitary-thyroid physiology, whereas semen volume, sperm count, motility, progressive motility, and morphology describe complementary aspects of the male partner’s reproductive profile [10,11]. A paired, couple-level analysis may therefore identify associations that are not visible when the two partners are considered in isolation. Such associations should not be interpreted as direct endocrine effects across partners; they may instead represent shared environmental, metabolic, behavioral, or unmeasured factors. This distinction is clinically important because established endocrine evidence largely concerns hormone concentrations within the male partner, and endocrine conditions in either partner can affect reproductive health through different pathways [12-15].

The source case-control study for the present analysis investigated vitamin D status in women with unexplained infertility and reported descriptive hormone and partner semen measurements [10]. The current manuscript uses the unexplained-infertility cohort only and addresses a distinct, previously unreported question: whether female FSH, LH, prolactin, E2, and TSH are associated with the semen parameters of their male partners.

The primary aim was to conduct a multiplicity-adjusted exploratory screen of female-hormone/partner-semen associations. The secondary aim was to examine whether selected signals persisted after adjustment for female age and BMI.

## Materials and methods

Study design, source population, and publication transparency

This was an exploratory cross-sectional secondary analysis of the 70 couples with unexplained infertility from a previously published case-control study at Ain Shams University Maternity Hospital, Egypt, conducted from October 2018 through April 2019 [10]. The original study enrolled 70 women with unexplained infertility and 70 fertile controls, primarily to evaluate vitamin D status. Its recruitment procedures, original eligibility criteria, clinical assessment, laboratory methods, and original vitamin D analyses are reported in full in the source publication [10].

The present analysis was limited to the 70 women with unexplained infertility and their male partners because semen parameters were available only for this paired cohort. It does not repeat the vitamin D case-control hypothesis. The new analyses comprise hormone-semen correlation screening, FDR adjustment, and adjusted regression models that were not reported in the original publication.

Variables

Female variables were age, BMI, infertility duration, type of infertility, FSH (mIU/mL), LH (mIU/mL), prolactin (ng/mL), E2 (pg/mL), and TSH (mIU/mL). Partner semen variables were semen volume (mL), sperm concentration (x10^6^/mL), total sperm count (x10^6^), total motility (%), progressive motility (%), and normal morphology (%). The supplied analytic dataset contained complete values for all variables used in the 70-couple analysis.

Statistical analysis

Statistical analyses were conducted in R version 4.3.3 (R Foundation for Statistical Computing, Vienna, Austria). Characteristics of the analytic cohort are shown as mean (standard deviation (SD)) and median (interquartile range (IQR)) to describe central tendency and distribution. The female-hormone/partner-semen screen consisted of 30 two-sided Spearman rank correlations. Raw p-values and Benjamini-Hochberg FDR q-values are reported.

Three targeted linear models were subsequently fitted: progressive motility modeled with prolactin, TSH, age, and BMI; morphology modeled with prolactin, TSH, age, and BMI; and sperm concentration modeled with E2, age, and BMI. Models used 5,000 nonparametric bootstrap resamples to estimate 95% percentile confidence intervals. Residual normality and variance inflation factors were assessed; the bootstrap intervals were retained as the primary interval estimates because of the modest sample size. No causal inference was intended. Two-sided p < 0.05 was treated as nominally significant; the FDR q values were emphasized for the exploratory correlation screen.

This report was prepared with reference to the Strengthening the Reporting of Observational Studies in Epidemiology (STROBE) guidance for cross-sectional observational analyses [11].

Ethics and consent

The source publication reports that written informed consent was obtained from participants and that the original protocol and related documents received ethical and research approval from the Council of the Obstetrics and Gynecology Department, Ain Shams University [10]. The authors confirm that they are authorized to undertake and submit this secondary analysis of the de-identified source dataset. There was no new participant contact or intervention.

Publication transparency statement

This manuscript uses the unexplained-infertility cohort from the prior publication by Ramy et al. [10]. The previous report evaluated vitamin D deficiency and included descriptive reporting of female hormone concentrations and partner semen characteristics. The present manuscript addresses a separate question and reports new female-hormone/partner-semen correlation screening, FDR adjustment, bootstrap analysis, and multivariable regression.

## Results

Analytic cohort

The secondary analysis included 70 couples with unexplained infertility. The mean female age was 27.80 years (SD 2.93), and the mean BMI was 24.66 kg/m^2^ (SD 2.02). Primary infertility was recorded in 43/70 couples (61.4%). Female and partner baseline measurements are shown in Table 1. These descriptive cohort values were reported in the original study and are reproduced here to characterize the analytic sample; the association analyses below are new.

**Table 1:** Characteristics of the 70-couple unexplained-infertility analytic cohort Values are mean (SD) and median (IQR), except infertility type. Baseline descriptive values were previously reported in the source study [4].

Characteristic	Mean (SD) or n/N (%)	Median (IQR)	Missing, n
Female age, years	27.80 (SD 2.93)	28.00 (26.00-30.00)	0
Female BMI, kg/m^2^	24.66 (SD 2.02)	25.00 (23.83-25.67)	0
Infertility duration, years	2.59 (SD 0.79)	2.50 (2.00-3.00)	0
Primary infertility	43/70 (61.4)	—	0
Secondary infertility	27/70 (38.6)	—	0
FSH, mIU/mL	7.60 (SD 0.92)	7.70 (6.93-8.00)	0
LH, mIU/mL	5.50 (SD 1.21)	5.60 (4.43-6.20)	0
Prolactin, ng/mL	13.10 (SD 4.78)	14.00 (9.15-17.17)	0
Estradiol (E2), pg/mL	55.30 (SD 9.78)	54.65 (47.72-62.40)	0
TSH, mIU/mL	1.86 (SD 0.45)	1.90 (1.50-2.10)	0
Partner semen volume, mL	2.93 (SD 0.63)	2.85 (2.62-3.10)	0
Partner sperm concentration, x10^6^/mL	19.68 (SD 10.34)	17.30 (16.92-18.05)	0
Partner total sperm count, x10^6^	57.72 (SD 36.89)	49.90 (45.70-55.13)	0
Partner total motility, %	54.60 (SD 4.48)	54.25 (51.82-56.30)	0
Partner progressive motility, %	41.49 (SD 5.15)	42.30 (38.35-44.60)	0
Partner normal morphology, %	66.41 (SD 8.60)	70.00 (65.17-72.17)	0

Exploratory correlation screen

Four of 30 female-hormone/partner-semen correlations remained below the FDR threshold. Prolactin was inversely correlated with progressive motility (rho = -0.472, raw p < 0.001, FDR q < 0.001) and normal morphology (rho = -0.595, raw p < 0.001, FDR q < 0.001). TSH was positively correlated with progressive motility (rho = 0.331, raw p = 0.005, FDR q = 0.038) and normal morphology (rho = 0.419, raw p < 0.001, FDR q = 0.003). E2 showed a nominal positive correlation with sperm concentration (rho = 0.284, raw p = 0.017), but its FDR q-value was 0.103. The complete pattern of all 30 correlations is displayed in Figure 1.

**Figure 1:**
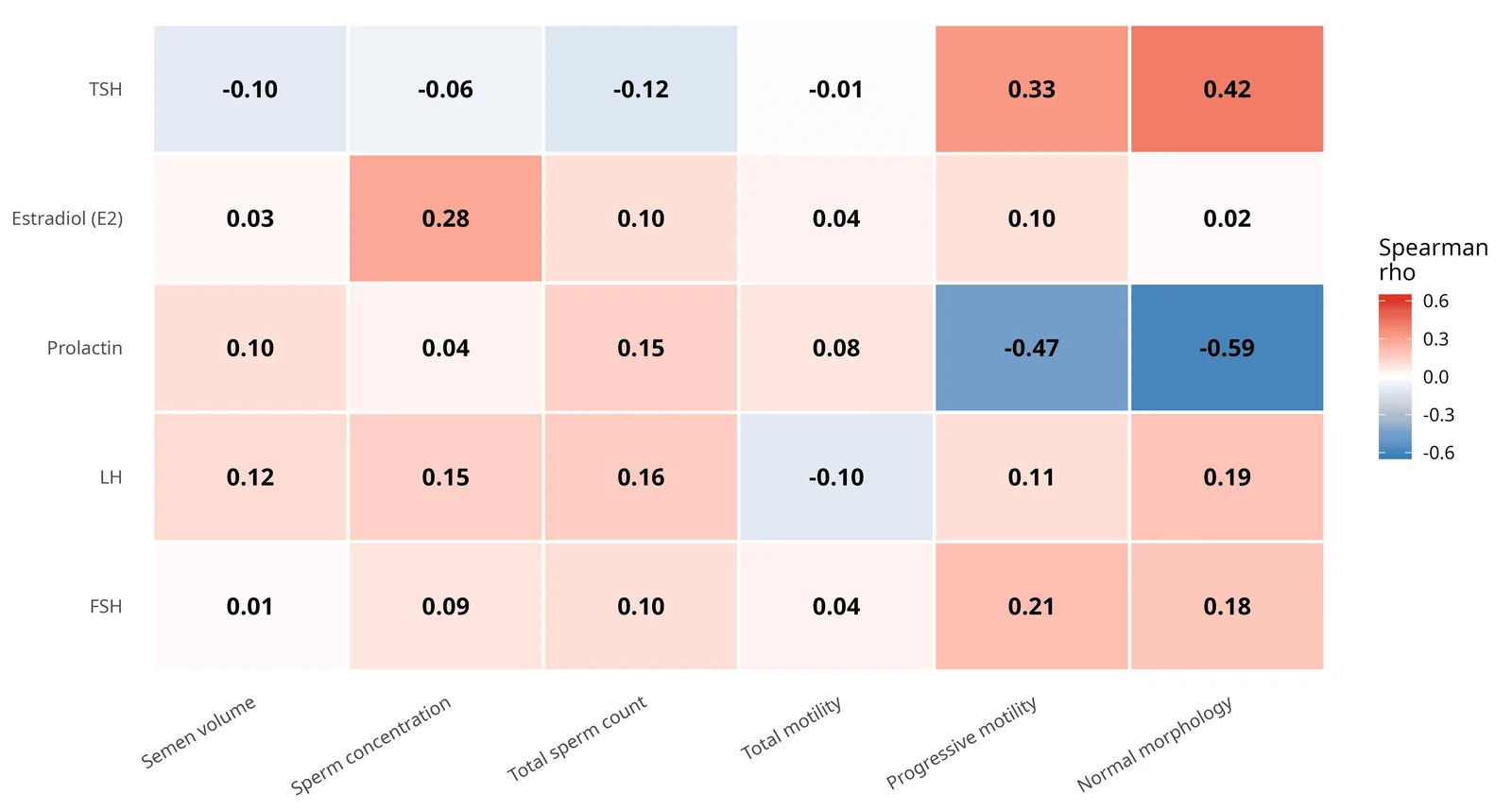
Full female-hormone/partner-semen correlation heatmap Spearman rho values are displayed for all 30 tested female-hormone/partner-semen associations in 70 couples. Color indicates the direction and magnitude of rho; darker blue indicates inverse correlations, and darker red indicates positive correlations. Statistical significance is not encoded within the figure; raw p-values and Benjamini-Hochberg FDR q-values are reported in Table 2. Source: Authors’ original figure, generated from the study dataset using R version 4.3.3 (R Foundation for Statistical Computing, Vienna, Austria) with ggplot2.

Table 2 presents the four FDR-significant associations together with the nominal E2/sperm-concentration association. Supplementary Table 1 reports the complete 30-test screen.

**Table 2:** Selected female-hormone/partner-semen Spearman associations in 70 couples The FDR q values are Benjamini-Hochberg adjusted across all 30 correlations. The E2/sperm concentration association is shown because it was nominally significant but did not survive FDR adjustment.

Female hormone	Partner semen parameter	n	Spearman rho	Raw p	FDR q
Prolactin	Normal morphology	70	-0.595	<0.001	<0.001
Prolactin	Progressive motility	70	-0.472	<0.001	<0.001
TSH	Normal morphology	70	0.419	<0.001	0.003
TSH	Progressive motility	70	0.331	0.005	0.038
E2	Sperm concentration	70	0.284	0.017	0.103

Adjusted models

The progressive-motility model explained 31.3% of outcome variance (adjusted R^2 ^= 0.271; model p < 0.001). Prolactin remained inversely associated with progressive motility after adjustment for TSH, age, and BMI (B = -0.401; bootstrap 95% CI -0.687 to -0.147; bootstrap p = 0.001). The TSH coefficient was positive, but its bootstrap confidence interval included zero (B = 2.560; bootstrap 95% CI -1.036 to 5.632).

The morphology model explained 38.1% of outcome variance (adjusted R^2 ^= 0.343; model p < 0.001). Prolactin remained inversely associated with morphology (B = -0.929; bootstrap 95% CI -1.282 to -0.620; bootstrap p < 0.001), and TSH remained positively associated (B = 4.076; bootstrap 95% CI 0.250 to 8.016; bootstrap p = 0.037). The adjusted E2 model for sperm concentration was not statistically informative (R^2 ^= 0.003; model p = 0.982). Table 3 presents all model coefficients.

**Table 3:** Targeted age- and BMI-adjusted linear models for partner semen parameters Bootstrap percentile confidence intervals and two-sided bootstrap p values are based on 5,000 resamples. Ordinary least-squares (OLS) p values are included for transparency but bootstrap inference is the primary reported interval analysis.

Outcome model	Predictor	Coefficient (B)	Bootstrap 95% CI	Bootstrap p	OLS p
Progressive motility	Prolactin	-0.401	-0.687 to -0.147	0.001	<0.001
Progressive motility	TSH	2.560	-1.036 to 5.632	0.199	0.042
Progressive motility	Age	-0.331	-0.751 to 0.117	0.147	0.084
Progressive motility	BMI	-0.250	-0.817 to 0.298	0.353	0.365
Normal morphology	Prolactin	-0.929	-1.282 to -0.620	<0.001	<0.001
Normal morphology	TSH	4.076	0.250 to 8.016	0.037	0.042
Normal morphology	Age	-0.115	-0.696 to 0.496	0.712	0.703
Normal morphology	BMI	0.032	-0.789 to 0.844	0.920	0.942
Sperm concentration	E2	-0.007	-0.245 to 0.164	0.998	0.956
Sperm concentration	Age	-0.082	-0.475 to 0.411	0.650	0.858
Sperm concentration	BMI	-0.200	-1.040 to 0.635	0.658	0.761

The four FDR-significant correlations are visualized in Figure 2.

**Figure 2:**
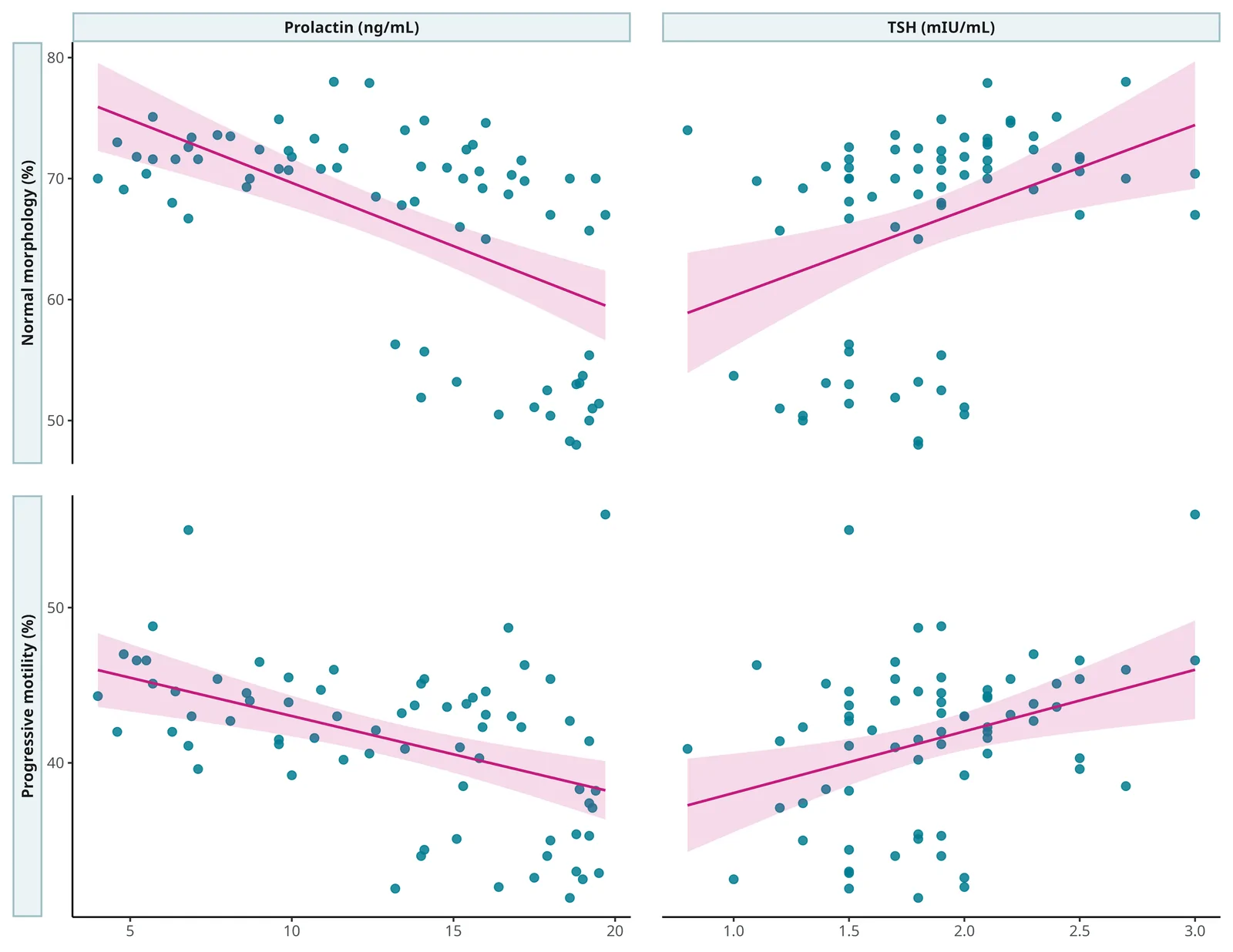
Focused female-hormone/partner-semen associations. Each point represents one couple. Columns display female prolactin and TSH, respectively; rows display partner normal morphology and progressive motility, respectively. Lines and shaded bands are descriptive ordinary linear-regression fits with 95% confidence bands. Statistical inference is based on the Spearman rank correlations and FDR q-values reported in Table 2, not on the fitted lines. Source: Authors’ original figure, generated from the study dataset using R version 4.3.3 (R Foundation for Statistical Computing, Vienna, Austria) with ggplot2.

## Discussion

Principal findings

In this exploratory secondary analysis of 70 couples with unexplained infertility, female prolactin had the most consistent cross-partner association with semen characteristics. Higher female prolactin was inversely associated with the male partner’s progressive motility and normal morphology in the FDR-adjusted screen and after adjustment for female age, BMI, and TSH. Female TSH was positively associated with partner morphology after adjustment, while its progressive-motility association was not robust to bootstrap inference. The nominal E2/sperm concentration association did not survive FDR adjustment and was not retained in the adjusted model.

Interpretation

These findings should not be interpreted to mean that a woman’s prolactin or TSH concentration directly changes her partner’s sperm quality. The analysis is cross-sectional and uses paired measurements at the couple level. The observed associations could reflect shared lifestyle, diet, environmental exposure, stress, metabolic health, healthcare-seeking patterns, residual confounding, or chance. The present results are best regarded as a signal for future paired-couple research, not as a basis for altered clinical testing or treatment.

The prolactin result should also be distinguished from evidence regarding male hyperprolactinemia. Elevated prolactin in men can adversely affect reproductive function through hypothalamic-pituitary-gonadal mechanisms, but that established male endocrine literature does not provide a mechanism or causal inference for a female partner’s prolactin concentration affecting semen [12]. Thyroid dysfunction has recognized reproductive implications in both sexes, although evidence concerning thyroid function and male infertility remains less definitive than the female reproductive literature [13,14]. In a prospective study of 145 presumed normal couples, male reproductive hormone profiles were associated with semen-quality measures but not with pregnancy status or time to pregnancy [15]. These established within-person endocrine findings cannot be extrapolated to a direct effect of one partner’s hormones on the other partner’s semen profile; they instead reinforce the need for cautious interpretation of the positive female TSH/male morphology association.

Current infertility guidance emphasizes joint assessment of both partners, including semen analysis in the male evaluation [1-3]. The current analysis extends that couple-level perspective methodologically. It does not, however, redefine unexplained infertility or establish an additional diagnostic pathway. Psychosocial stress, quality of life, and socio-behavioral factors have been associated with semen quality in reviews, and these factors can be shared within couples [16,17]. Likewise, evidence syntheses associate male adiposity, cigarette smoking, and alcohol intake with semen-quality measures [18-20]. The influence of physical activity, environmental toxicants, and diet has also been examined, although the underlying evidence is heterogeneous and does not permit causal interpretation of the present cross-partner findings [21-23]. Together, these factors provide plausible alternative explanations for the observed correlations.

Strengths and limitations

Strengths include the use of paired hormone and semen data in a defined unexplained-infertility cohort, complete analytic data for all 70 couples, reporting of the entire 30-association screen, FDR adjustment, bootstrap confidence intervals, and transparent separation of new analyses from the original vitamin D study.

The study has important limitations. It was a retrospective secondary analysis with a modest sample size and no prospectively prespecified hormone-semen hypotheses. The cross-sectional design prevents assessment of temporal sequence or causality. The source cohort excluded recognized male-factor infertility and had a single semen assessment per male partner, which may have limited outcome variability and generalizability. Data on male reproductive hormones, repeated semen samples, shared lifestyle exposures, medications, relationship factors, and subsequent pregnancy outcomes were unavailable for this analysis. This is important because conventional semen analysis has recognized limits as a fertility test, and repeated analyses show within-person variability [7,24]. A contemporary cohort likewise concluded that a single sample may be insufficient to characterize semen abnormalities consistently [25]. Although FDR adjustment was used for the correlation screen, residual false-positive risk remains.

Future research

Future prospective studies should prespecify couple-level hypotheses, collect repeated semen analyses, measure relevant endocrine variables in both partners, document shared lifestyle and environmental exposures, and evaluate whether paired endocrine/semen patterns are associated with fecundability, treatment response, or live birth. Independent replication in larger and more diverse populations is essential before these associations can inform clinical care.

## Conclusions

In this exploratory secondary analysis of 70 couples with unexplained infertility, female prolactin was consistently inversely associated with partner progressive sperm motility and normal morphology. Female TSH showed a positive association with partner morphology, while the nominal E2/sperm concentration association was not robust after multiple-testing adjustment and covariate adjustment. These couple-level findings are hypothesis-generating and require independent prospective replication.

## Appendices

Publication transparency statement

This manuscript uses the unexplained-infertility cohort from the prior publication by Ramy et al. [10]. The previous report evaluated vitamin D deficiency and included descriptive reporting of female hormone concentrations and partner semen characteristics. The present manuscript addresses a separate question and reports new female-hormone/partner-semen correlation screening, FDR adjustment, bootstrap analysis, and multivariable regression.
